# Blood pressure control in patients with a previous stroke/transient ischaemic attack in primary care in Ireland: a cross sectional study

**DOI:** 10.1186/s12875-020-01211-z

**Published:** 2020-07-10

**Authors:** Róisín Doogue, David McCann, Noirin Fitzgerald, Andrew W. Murphy, Liam G. Glynn, Peter Hayes

**Affiliations:** 1 Graduate Entry Medical School, University of Limerick, Limerick, Ireland; 2 Department of General Practice & HRB Primary Care Clinical Trial Network Ireland, National University of Ireland Galway, Galway, Ireland; 3 Health Research Institute, University of Limerick, Limerick, Ireland

**Keywords:** Blood pressure guidelines, Dosing, Hypertension, Prevalence, Primary care, Stroke

## Abstract

**Background:**

Uncontrolled blood pressure (BP) is an important modifiable risk factor for recurrent stroke. Secondary prevention measures when implemented can reduce stroke re-occurrence by 80%. However, hypertension control rates remain sub-optimal, and little data is available from primary care where most management occurs.

The aim of this study was to describe BP control in primary care-based patients with a previous stroke or transient ischaemic attack (TIA) in Ireland, and to concurrently examine antihypertensive medication-dosing.

**Methods:**

Study participants most recent office-based BP reading was compared with the NICE (NG136) and European Society of Hypertension/ European Society of Cardiology (ESH/ESC 2013) goal of BP < 140/90 mmHg. Optimal anti-hypertensive medication dosing was determined by benchmarking prescribed doses for each drug with the World Health Organisation-Defined Daily Dosing (WHO-DDD) recommendations.

**Results:**

We identified 328 patients with a previous stroke or TIA in 10 practices. Blood pressure was controlled in almost two thirds of patients when measured against the ESH/ESC and NICE guidelines (63.1%, *n* = 207). Of those with BP ≥140/90 (*n* = 116), just under half (*n* = 44, 47.3%) were adequately dosed in all anti-hypertensive medications when compared with the WHO-DDD recommendations.

**Conclusion:**

Blood pressure control in patients post stroke/TIA appears sub-optimal in over one third of patients. A comparison of drug doses with WHO-DDD recommendations suggests that 47% of patients may benefit from drug-dose improvements. Further work is required to assess how best to manage blood pressure in patients with a previous stroke or TIA in Primary Care, as most consultations for hypertension take place in this setting.

## Introduction

Stroke has a major impact on people’s lives, with often devastating personal, social and economic consequences for the individual and their family. The cost of stroke in the European Union (EU) in 2015 was estimated at €45 billion, accounting for a total mortality rate of 17% within the EU, making it the second most common cause of death [1]. Increased disability and mortality rates result from recurrent strokes, yet despite this, an assessment of the availability of secondary prevention measures after stroke or transient ischaemic attack (TIA) across Europe has shown significant gaps in specialist care, monitoring and treatment programmes [2]. The European Stroke Action Plan (ESAP) for the years 2018–2030 outlined targets for the development of stroke care [3]. The report outlined six domains in their action plan, one of which is secondary prevention and organised follow-up. The 5-year risk of recurrent stroke is estimated at 9.5% with recurrent stroke often resulting in more debilitating outcomes [4].

Management of risk factors such as smoking, hyperlipidaemia, obesity, diabetes, atrial fibrillation, sedentary lifestyle, raised body mass index and hypertension, have the potential to reduce recurrent events by up to 80% [3, 5]. Of these, researchers have demonstrated hypertension to be the most important modifiable risk factor in stroke [6]. In recurrent stroke the risk increases by about one-third for every 10 mmHg increase in systolic blood pressure [7].

In a survey of secondary prevention of stroke in Europe, satisfactory levels of blood pressure (BP) control are achieved in less than 60% of countries [2]. Figures from Ireland were included in this data. However, there were limitations to this study. Authors didn’t have access to primary registry data and many of the responses were estimated, allowing for the possibility of unintentional biases. A recent paper published in the Lancet showed that Ireland, Finland and Spain have the lowest rate of awareness, treatment and control of BP in their populations, based on an analysis of national representative surveys in 12 high-income countries [8].

Reasons for sub-optimal BP control are multi-faceted and include patient factors (adherence) [9, 10], physician factors (including therapeutic inertia) [10], lifestyle issues and treatment resistant hypertension [11]. However, a recent study considering pseudo-resistance in high-risk cardiovascular patients suggests that treatment resistant hypertension may be less prevalent than expected, with half of the patients in this study prescribed sub-optimal doses of their anti-hypertensive medications [12].

Blood pressure guidelines for the prevention of stroke have been the subject of much discussion with differences emerging between specialist groups. The recent American Heart Association (AHA) guidelines have adopted a target of < 130/80 mmHg for the secondary prevention of stroke [13]. The European Society of Cardiology/ European Society of Hypertension (ESC/ ESH) changed their guidance from < 140/90 mmHg [14] to < 130/80 mmHg in their most recent guidelines published in 2018 [11]. Recent hypertension guidelines from the National Institute of Health and Care Excellence (NICE) have not committed to the lower target and instead have set a target of < 140/90 mmHg for adults under 80 years [15, 16].

The Irish health care system does not have universal registration with a GP or mandatory coding of diseases. Almost half of the population is registered through the Primary Care Reimbursement Service (PCRS) and registered to one single general practitioner [17]. The remainder are described as private patients and able to see any general practitioner. All patients over the age of 70 years, under six, and those below defined income levels [less than €304 (£270) gross per week for a single person; €441 (£395) gross per week for a couple] are registered with the PCRS [18]. Of those registered with the PCRS- three quarters receive free primary healthcare and medications (those with the lowest income), the remaining quarter have free access to primary care only [19]. We based all prevalence analysis on patients who are registered with the PCRS as we can be certain these patients are registered with a single practice.

The primary aim of this study was to describe the prevalence of sub-optimal blood pressure control in a cross-sectional cohort of patients with a previous stroke/ TIA in primary care. Secondly, we wished to examine the characteristics of this cohort- looking for associations that could predict poor blood pressure control and thirdly we wished to assess anti-hypertensive drug dosing schedules in these patients. To our knowledge, this is the first study examining these issues in primary care.

## Methods

General practices in the University of Limerick, Education and Research Network [20] were invited to participate via email. Out of the 14 practices that responded, ten practices were selected as they were within 2 h travel from the university, had the capacity to host the researcher and used electronic health care records. The 10 practices that participated included a variety of practice sizes (small, medium, large) and types (urban, rural, mixed, teaching and non-teaching) (Table 1). This was a convenience sample but the participating practices were purposefully selected to be representative of Irish General Practice [21].

**Table 1:** General practice characteristics

Practice Type		Practice Location		Practice Size		Involved in post graduate GP training	
Single Handed	(*n* = 2)	Urban	(*n* = 2)	< 1000 patients	(*n* = 5)	Yes	(*n* = 3)
2–3 GPs	(*n* = 6)	Rural	(*n* = 4)	1000–2000 patients	(*n* = 3)	No	(*n* = 7)
> 3 GPs	(*n* = 1)	Mixed	(*n* = 4)	> 2000 patients	(*n* = 2)		

Data collection was conducted in July and August 2019 according to a prespecified data extraction protocol designed by RD (Appendix). Data collection was supported in practices by DMC, who received a summer student research scholarship, and was supervised by RD. This work fulfilled for the GP, the Irish Medical Council requirement to conduct an annual audit.

Eligible patients were identified as those patients who were coded for stroke or TIA or those who were not coded but had a hospital diagnosed stroke or TIA in their discharge summaries from specialists. These events were classed as either ischaemic stroke, haemorrhagic stroke, TIA or unknown. If patients had more than one of these events, they were classed based on their most severe event. Disease coding typically categorised the type of each stroke, where this was not available or ambiguity existed, hospital discharge summaries and consultant reports were examined to clarify the diagnosis.

All practices used electronic health care records, but used a variety of software systems; Socrates®, Health One™ or Helix Practice Manager. Search methods differed between the systems, some using specific diagnostic searches and others disease coding, each using the analysis function inbuilt in that system.

In Socrates®, the International Classification of Disease-10 (ICD-10) [22] codes G45, and I60-I64 were used, along with the International Classification of Primary Care-2 (ICPC-2) [23] specific codes K89 (Transient cerebral ischaemia), K90 (Stroke/cerebrovascular accident), and K91 (Cerebrovascular disease) to facilitate case identification. In Health One® and Helix Practice Manager they searched using keywords, such as “stroke, cerebrovascular accident, CVA, infarct, cerebral bleed, subarachnoid haemorrhage, TIA, Transient Ischemic attack” in the patient’s past history section if coding of disease was not used in that practice.

Sub-optimal BP control was assessed by comparing the last office BP on file with the ESH/ESC 2013 [14] and NICE guideline (NG136) [16] recommendation of BP < 140/90 mmHg for the secondary prevention of stroke. Only patients who were actively attending the general practitioner were included. European guidelines are more applicable to the Irish context and at the time of the study Irish GPs would have been more familiar with the 2013 ESH/ESC guideline [14] or the NICE guidelines [24]. In the absence of an up to date Irish guideline, the most recent published in 2010 [25], Irish GPs are more likely to follow European and NICE guidelines [24] and therefore we used BP < 140/90 mmHg as the target.

To help describe the characteristics of these patients we recorded, if available, demographics, stroke subtype, blood pressure, kidney function, lipid profile and drug data. Co-morbidities of diabetes and chronic kidney disease (CKD) were also recorded. Patients were described as having diabetes if they had ICPC codes T89 (Diabetes insulin dependent) or T90 (Diabetes non-insulin dependent), ICD codes E10 (Type 1 Diabetes Mellitus), E11 **(**Type 2 Diabetes Mellitus), patient’s active diagnosis contained diabetes keyword, or were taking insulin or oral hypoglycaemic agents. Patients were described as having CKD if an estimated Glomerular Filtration Rate (eGFR) less than 60mls/ min/ 1.73m^2^ was recorded in the last two renal function tests [26]. Anti-hypertensive medications were recorded as defined by the British National Formulary (BNF) 2019 [27], and included the following drug groups: diuretics, angiotensin converting enzyme (ACE) inhibitors, calcium channel blockers, angiotensin II type 1 receptor blockers, adrenergic beta antagonists and doxazosin.

The use of ambulatory blood pressure monitoring (ABPM) was examined, especially in those with sub-optimal BP control to identify cases of white coat hypertension (a normal 24-h ABPM, with an elevated manual office BP reading). Day time average readings > 134/84 mmHg signified sub-optimal BP control [11].

Optimal anti-hypertensive medication dosing use was determined by benchmarking prescribed anti-hypertensive drug doses, for each individual prescribed drug, against the World Health Organisation-Defined Daily Dosing (WHO-DDD) schedule. We used this as a surrogate for investigating the adequacy of dosing. The WHO-DDD is the assumed average maintenance dose per day for a drug, used for its main indication in adults [28]. Egan et al. adopted a slightly different approach, examining the number of patients who were receiving for each medication, diuretics apart, at least half the maximum dose [29]. We present both approaches here. Patients were deemed to be adequately dosed when all prescribed drugs were at or above these thresholds.

Graphical summaries were created for all patient characteristics to identify any anomalies or potential outliers. Summary statistics were generated that were appropriate for the explanatory variable in question (i.e. mean, standard deviation and medians). Independent t-tests were used to compare the mean of the various quantitative variables between the groups, controlled (< 140/90 mmHg) and uncontrolled (≥140/90 mmHg) and a two-sample comparison of proportions (chi-square test) to similarly compare factors. Chi-squared tests were also used to test whether a significant difference was found between the expected frequencies in different categories in controlled (< 140/90 mmHg) versus uncontrolled (≥140/90 mmHg) patients. Binary logistic regression was used to model the association between the (log) odds of BP control (< 140/90 mmHg) and explanatory variables of interest. All statistical analysis took place in SPSS- version 26 [30].

## Results

Ten practices participated (Table 1) with 328 patients identified as having a previous stroke or TIA. When office BP readings were compared with the ESH/ ESC 2013 [14] and NICE (NG136) [16] guidelines (< 140/90 mmHg), we found that 116 (35.9%) patients had sub-optimal BP control.

Patient characteristics are described in Table 2. 62.2% of the population (*n* = 328) studied are male with 37.8% female. Stroke subtype included 29.3% ischaemic stroke, 9.8% haemorrhagic stroke and 41.2% TIA, with 19.8% unknown or not recorded. 87.8% were registered with PCRS for free GP services. Multimorbidity is a common feature within this cohort of patients, 15.5% had diabetes, 22.6% had CKD and 4.9% had both diabetes and CKD.

**Table 2:** Patient characteristics by blood pressure

	BP < 140/90	BP ≥ 140/90	no BP recorded	Total	% of Total
Total	207 (63.1%)	116 (35.4%)	5 (1.5%)	328	–
Female	71 (34.3%)	50 (43.1%)	3 (60%)	124	37.8%
Male	136 (65.7%)	66 (56.9%)	2 (40%)	204	62.2%
Average age (SD)	72.8 (12.3)	74 (10.5)	70.8 (8.8)	73.3 (11)	–
**Stroke subtype:**					
~ Ischaemic	57 (27.5%)	38 (32.8%)	1 (20%)	96	29.3%
~ Haemorrhagic	21 (10.2%)	10 (8.6%)	1 (20%)	32	9.8%
~ TIA	84 (40.6%)	50 (43.1%)	1 (20%)	135	41.2%
~ Unknown	45 (21.7%)	18 (15.5%)	2 (40%)	65	19.8%
**GMS Status:**					
~ Full Medical card	152 (73.4%)	93 (80.2%)	5 (100%)	250	76.2%
~ Doctor visit card	26 (12.6%)	12 (10.3%)	0 (0%)	38	11.6%
~ Private patient	29 (14%)	11 (9.5%)	0 (0%)	40	12.2%
**Multimorbidity:**					
~ Diabetes	32 (15.5%)	19 (16.4%)	0 (0%)	51	15.5%
~ CKD	44 (21.3%)	29 (25%)	1 (20%)	74	22.6%
~ Diabetes & CKD	13 (6.3%)	6 (5.2%)	0 (0%)	19	4.9%
~ Total Chol > 4.5	50 (17.6%)^a^	37 (13%)^a^	2 (0.7%)^a^	89^a^	31.3%^a^
~ LDL > 2.5	54 (19.8%)^a^	35 (12.8%)^a^	0 (0%)^a^	89^a^	32.6%^a^
**Drugs prescribed:**					
~ aspirin	95 (45.9%)	64 (55.2%)	2 (40%)	161	49.1%
~ clopidogrel	37 (17.9%)	19 (16.4%)	0 (0%)	56	17.1%
~ statin	154 (74.4%)	89 (76.7%)	3 (60%)	246	75%
~ warfarin	44 (21.3%)	12 (10.3%)	0 (0%)	56	17.1%
~ DOAC	14 (6.8%)	8 (6.9%)	0 (0%)	22	6.7%
**ABPM ever**	95 (45.9%)	64 (55.2%)	0 (0%)	159	48.5%

^a^Total cholesterol and LDL assessed in a subset of patients. Total cholesterol (*n* = 284), LDL (*n* = 273)

Independent t-tests were used to compare the mean of the various quantitative variables (age, parameters of renal function, ambulatory blood pressure, lipids) between the two groups, controlled (< 140/90 mmHg) and uncontrolled (≥140/90 mmHg). A two-sample comparison of proportions (or chi-square test) to compare factors (gender, PCRS status, co-morbidity and sub-optimal BP control (≥140/90 mmHg) was also performed. No statistically significant differences between the groups were demonstrated.

Chi-squared tests were also used to test whether a significant difference was found between the expected frequencies and the observed frequencies in different categories in controlled (< 140/90 mmHg) versus uncontrolled patients (≥140/90 mmHg), and no significant differences were observed in sex, stroke subtype, GMS status and comorbidities. Binary logistic regression was used to model the association between the (log) odds of BP control and explanatory variables of interest. No predictors of sub-optimal BP were identified.

Detailed data on drug-dosing was available for 252 patients prescribed antihypertensive medications (Table 3). For those with BP ≥140/90 mmHg, 44 (47.3%) patients and 22 (23.7%) patients were inadequately dosed, in each of their medications, according to WHO-DDD guidelines [28] and Egan et al. guidelines [29] respectively. Of the 116 patients that had uncontrolled BP ≥140/90 mmHg, 23 patients were not taking any anti-hypertensive medication and 31 were taking a single agent.

**Table 3:** Antihypertensive dosing by blood pressure

	BP < 140/90 *n* = 207	BP ≥ 140/90 *n* = 116	No BP recorded *n* = 5	Total *n* = 328
0 antihypertensives prescribed:	51 (24.6%)	22 (19%)	2 (40%)	73 (22.3%)
1 antihypertensive prescribed	65 (31.4%)	31 (26.7%)	1 (20%)	97 (29.6%)
**Detailed analysis of antihypertensives prescribed**				
Patient on ≥1 antihypertensive	*n* = 156	*n* = 93	n = 3	*n* = 252^a^
Adequate dose (WHO)	73 (46.8%)	49 (52.7%)	1 (33.3%)	123 (48.8%)
Inadequate dose (WHO)	83 (53.2%)	44 (47.3%)	2 (66.6%)	129 (51.2%)
Adequate dose (Egan)	105 (67.3%)	71 (76.3%)	1 (33.3%)	177 (70.2%)
Inadequate dose (Egan)	51 (32.7%)	22 (23.7%)	2 (66.6%)	75 (29.8%)

^a^Antihypertensive dose not recorded for one patient

Results of ABPM were available for 64 (55.2%) of the 116 patients with sub-optimal BP control. Of those who had ABPM (*n* = 64), nine patients demonstrated white coat hypertension, the remaining 55 had sub-optimal BP control, with average day time readings > 134/84 mmHg [11, 14].

## Discussion

### Summary of main findings

Blood pressure control was sub-optimal in approximately one-third (35.4%) of patients according to ESH/ESC 2013 and NICE (NG136) guidelines, where BP < 140/90 mmHg is deemed satisfactory. Anti-hypertensive medication dosing appears sub-optimal in close to half of these patients when compared with WHO-DDD criteria.

### Comparison with existing literature

Of those identified with a previous stroke or TIA, 63.1% had blood pressure controlled to < 140/90 mmHg. This compares well with results from a study in Norwegian general practice finding that 47% of patients, 1 year post stroke, had BP controlled to < 140/90 mmHg [31]. However, there is a paucity of research examining blood pressure control and secondary prevention of stroke in the primary care setting.

Due to an aging population, the number of people with stroke is set to rise by 58% between 2007 and 2021 [32]. This projected rise in the number of strokes will have a significant impact on health care spending. The cost of stroke has been estimated at €557 million per annum in Ireland [33], €4.11 billion (£3.6 billion) per annum in the UK [34] and €45 billion per annum within the EU [1]. Consequently, with rising health care costs, it is imperative that prevention measures reducing the incidence of stroke or further strokes, are adopted.

For clinicians, deciding which is the most appropriate target for blood pressure control in this group of patients is difficult. Having two or three differing guidelines, each recommending different targets can cause confusion regarding the most appropriate clinical management. The United Kingdom, National Institute for Health and Care Excellence (NICE- Clinical Guideline NG136) [16] has recently reviewed the evidence and decided that the current target of < 140/90 mmHg shall be maintained for all adults under 80 years [16], and will not follow the AHA guidelines to reduce blood pressure targets to < 130/80 mmHg, as evidenced by the SPRINT trial [35]. NICE stated that the methodology used to measure BP in SPRINT is simply not achievable in clinical care settings at this stage [36]. The current ESC/ESH guideline [11] has changed its recommendation from a target BP of < 140/90 mmHg [14] (as used in our study) to < 130/80 mmHg. However, it recommends caution with lower targets, especially in those patients over 65 years where the target is 130–140/80 mmHg, if tolerated. Caution must be exercised with all patients, as reducing systolic blood pressure to < 120 mmHg may provide benefit for some persons, but problems for others [37].

Achieving blood pressure targets in this complex co-morbid group of patients – with a mean age of 73 years, and in whom a quarter have CKD - can be difficult. There are occasions in practice when meeting blood pressure guidelines is not appropriate and higher blood pressure readings are acceptable– those with concurrent unstable coronary artery disease, those with previous anti-hypertensive based acute kidney injury or cerebral hypo-perfusion. A further group worth mentioning are those with extreme variability in blood pressure readings (mixed high and low) or those with isolated systolic hypertension and a large pulse pressure. These groups cannot often tolerate focused blood pressure reduction programs and treating physicians do not pursue further after trial of treatment, as this can impact seriously on their quality of life. We accept this may be a reality for some patients.

Interestingly, of those with uncontrolled BP ≥140/90 mmHg (*n* = 116), 22 patients did not have any anti-hypertensive medications prescribed and a further 31 patients had only a single agent prescribed. Current ESC/ESH guidelines advise that two anti-hypertensive agents are required to ensure BP control in most instances, and these should be commenced initially as a fixed dose combination therapy [11].

Of the 93 patients prescribed one or more antihypertensives, 47.3% (*n* = 44) of these did not meet the recommended dose in all their anti-hypertensive drugs when measured against the WHO-DDD guidelines (Table 3). The WHO-DDD is a stringent measure of the adequacy of dosing, we accept this. However to counter balance we also analysed drug dosing from a methodology used by Egan et al. [29]. Here, where the threshold for dosing adequacy is much lower, almost a quarter of patients are still inadequately dosed when BP remains > 140/90 mmHg.

It is acknowledged that a patient with a clear indication for drug treatment may sometimes not be prescribed the drug due to factors like liver or kidney failure, weight, patient receiving end of life care, and/or previous adverse response to the drug in question. It can be accepted that in some instances, not receiving a drug treatment does not necessarily suggest poor medical treatment or therapeutic nihilism. However, as Gil-Guillen et al. suggest, physician inertia to escalate drug-dosing in hypertension can be a significant problem up to 70% of the time [38].

The ESC/ESH guidelines support the use of ABPM when investigating high office blood pressure readings [11]. It is an important tool when investigating white coat hypertension. It also provides important information on night time BP control which is an independent predictor of death and negative cardiovascular outcomes [39]. Nevertheless, providing ABPM in general practice can be costly and is not suitable for all patients e.g. those with pulse irregularity such as atrial fibrillation, those who are easily confused or those who find it too uncomfortable to wear. Results from this study show that ABPM could be utilised to a greater extent as just over half the cohort have ever had ABPM. Reimbursement for the use of ABPM by primary care practitioners in Ireland, is available since 2018 for all PCRS registered patients. It is anticipated that this will lead to an increase use of ABPM as a tool for diagnosis and monitoring into the future.

### Strengths and limitations

We believe this is the first estimate of blood pressure control in a cohort of patients post stroke/TIA in primary care with specific consideration of anti-hypertensive drug dosing. A further strength of this study was that all data was collected at the level of the patient’s file in their own general practice.

Limitations of this study include the use of 10 general practices from the same geographical area. The cross-sectional design took in to account BP readings from one point in time only and did not take in to consideration out of office BP monitoring or indeed the other factors that may influence recurrent stroke (smoking, hyperlipidaemia, obesity, diabetes, atrial fibrillation and sedentary lifestyle). As this was a retrospective study, there was no common protocol for the measuring and recording of office BP. It is also accepted that the WHO-DDD is a surrogate marker for assessing the adequacy of drug-dosing.

### Implications for research and practice

The results of this study have provided important information on blood pressure control in patients who have had a previous stroke or TIA. Recent stroke audits have focused primarily on acute hospital care [4, 40, 41]. There is a paucity of research in primary care. Community care and long term follow up in primary care has been underfunded and under-resourced, despite recommendations from recent stroke audits to health authorities to address this deficit [40]. Gaps in secondary prevention could be tackled through EU-wide policies, development of national and regional guidelines and strategies, and direct intervention-based reimbursement [2].

A new contract for general practice services in Ireland has been negotiated recently [42]. This aims to provide essential resources and funding to build the capacity for chronic disease management in primary care. Care for people who have had a cerebrovascular event (stroke or TIA) is addressed as part of this plan. Providing funding for chronic disease management is a recent advancement in primary care in Ireland. It has been available for the care of people with Type 2 Diabetes who are registered with PCRS since 2017. It has benefited these patients by improving and standardising the recording of clinical information and has also shown an improvement in the achievement of clinical targets [43].

There is clear evidence that reducing blood pressure to below 140/90 mmHg benefits those who have had a previous stroke or TIA [11]. This BP level should be the desired initial target for patients and their doctors before considering further reductions. Further research is needed to explore how GPs might be supported to optimise the management of BP. This may involve an examination of prescriber inertia to increase anti-hypertensive drug doses, supporting the use of out-of-office readings to confirm sub-optimal control and enhance BP management, and patient strategies to encourage adherence to drug therapy and the adoption of a healthy lifestyle. These challenges may be met by introducing novel approaches for BP control in primary care, such as home care and self-care approaches [44].

## Conclusion

Blood pressure control appears sub-optimal in at least one-third of patients with a previous stroke or TIA. Half of these patients could respond to anti-hypertensive dose escalation. Further work is required to see how best to manage blood pressure in patients with a previous stroke or TIA in Primary Care, as the majority of consultations for hypertension take place in this setting.

## Data Availability

The datasets used and/or analysed during the current study are available from the corresponding author on reasonable request.

## Appendix

### Data extraction protocol

Practice No. (P1 or P2) followed by patient no. (i.e. 001–1000-e.g. P1001, or P2001)

Gender (1 = Female 2 = Male)

GMS (0 = Private 1 = GMS 2 = DVC card)

Age in yrs.

Coded TIA (1 = yes 0 = No) ICD-10 G45, ICPC-2 K89

Coded Stroke (1 = yes 0 = No) ICD-10 I60-I64, ICPC-2 K90–91

Coded Ischaemic stroke (1 = yes 0 = No) ICD-10 I63–64, ICPC-2 K90–91

Coded Haemorrhagic stroke (1 = yes, 0 = no) ICD-10 I60-I62, ICPC-2 K90

Non-coded Key words (stroke, cerebrovascular accident, CVA, infarct, cerebral bleed, subarachnoid haemorrhage, TIA, Transient Ischemic attack

Non-coded TIA identified through chart search. Personal knowledge, medication search, cross referencing etc. (1 = yes 0 = No)

If yes, how identified: Add free text

Non-coded STROKE identified through chart search. Personal knowledge, medication search, cross referencing etc. (1 = yes 0 = No)

If yes, how identified: Add free text

Most Recent Creatinine in mmols/L

Next most Recent Creatinine in mmols/L

Date of Most Recent Creatinine (DD/MM/YYYY)

Date of next most Recent Creatinine (DD/MM/YYYY)

Most Recent eGFR-calculate if needs be

Next most Recent eGFR-calculate if needs be

Date of Most Recent eGFR (DD/MM/YYYY)

Date next Most Recent eGFR (DD/MM/YYYY)

Most recent Total Cholesterol

Most recent LDL

Date of most recent Total Cholesterol and LDL

Date of last Recorded manual Blood Pressure if available (DD/MM/YYYY)

Systolic Blood Pressure most recent in mmHg

Diastolic Blood Pressure most recent in mmHg

Date of last Recorded 24-h ABPM if available (DD/MM/YYYY)

Daytime average ABPM Systolic Blood Pressure in mmHg

Daytime average ABPM Diastolic Blood Pressure in mmHg

Nocturnal average ABPM Systolic Blood Pressure in mmHg

Nocturnal average ABPM Diastolic Blood Pressure in mmHg

24 h average ABPM Systolic Blood Pressure in mmHg

24 h average ABPM Diastolic Blood Pressure in mmHg

Anti-hypertensive medication. Reference BNF 77: March–September 2019.

Anti- hypertensive med 1 name

Anti- hypertensive med 1 dose

Anti- hypertensive med 2 name

Anti- hypertensive med 2 dose

Anti- hypertensive med 3 name

Anti- hypertensive med 3 dose

Anti- hypertensive med 4 name

Anti- hypertensive med 4 dose

Aspirin (1 = yes, 0 = no)

Plavix (yes/no)

Other anti-platelet (list name and dose)

Statin (1 = yes, 0 = no)

DOAC (1 = yes, 0 = no)

Name of DOAC

Warfarin (1 = yes, 0 = no)

## Abbreviations

ABPM: Ambulatory Blood Pressure Monitoring
AHA: American Heart Association
BP: Blood Pressure
CKD: Chronic Kidney Disease
eGFR: Estimated Glomerular Filtration Rate
ESAP: European Stroke Action Plan
ESC: European Society of Cardiology
ESH: European Society of Hypertension
EU: European Union
HBPM: Home Blood Pressure Monitoring
ICD-10: International Classification of Disease-10
ICPC-2: International Classification of Primary Care-2
NICE: National Institute for Health and Care Excellence
PCRS: Primary Care Reimbursement Service
TIA: Transient Ischaemic Attack
WHO-DDD: World Health Organisation-Defined Daily Dosing
